# The Influence of Game Intervals on Physical Performance Demands in Elite Futsal: Insights from Congested Periods

**DOI:** 10.3390/sports13020056

**Published:** 2025-02-14

**Authors:** Augusto Pereira, João Nuno Ribeiro, Pedro E. Alcaraz, Rubén Herrero Carrasco, Bruno Travassos, Tomás T. Freitas, Konstantinos Spyrou

**Affiliations:** 1 UCAM Research Center for High Performance Sport, UCAM Universidad Católica de Murcia, 30107 Murcia, Spain; augusto.pereiram23@gmail.com (A.P.); palcaraz@ucam.edu (P.E.A.); tfreitas@ucam.edu (T.T.F.); 2 Department of Sport Sciences, Universidade da Beira Interior, 6200 Covilhã, Portugal; joaonunorib@gmail.com (J.N.R.); bfrt@ubi.pt (B.T.); 3 School of Education, Communication and Sports, Polytechnic Institute of Guarda, 6300 Guarda, Portugal; 4 SPRINT Sport Physical Activity and Health Research & Innovation Center, 2040 Rio Maior, Portugal; 5 Facultad de Deporte, UCAM Universidad Católica de Murcia, 30107 Murcia, Spain; 6 Strength & Conditioning Society, 30008 Murcia, Spain; 7 Faculty of Sports, Universidad Internacional Isabel I de Castilla, 09003 Burgos, Spain; rubenhffrm@gmail.com; 8 Research Center in Sports Sciences, Health Sciences and Human Development (CIDESD), 6201 Covilhã, Portugal; 9 Portugal Football School, Portuguese Football Federation, 1495 Oeiras, Portugal; 10 NAR-Nucleus of High Performance in Sport, São Paulo 04753-060, Brazil

**Keywords:** external load, five-a-side soccer, futsal, performance, team sports

## Abstract

The aims of this study were to analyze (1) the external match demands during a congested period (CP) (i.e., three games in eight days) and (2) the differences among games with two- or three-day intervals in professional futsal players. Eleven elite male futsal players were monitored during 15 official matches. Wearable accelerometers were used to record player load (PL), accelerations (ACC), decelerations (DEC), and changes of direction (COD) at different intensities (e.g., high, medium, and low) using two approaches (e.g., absolute and relative per minute). A linear mixed model and effect sizes (ESs) were used to analyze differences between matches and days of interval. Considering the external match load during CP, non-significant differences were found for all the variables (*p* = 0.108–0.995; ES: 0.01–0.40). Comparing the interval days between games, players had significantly higher DEC_HI_ (*p* = 0.030; ES: 0.48), COD_TOTAL_ (*p* = 0.028; ES: 0.33), COD_MED_ (*p* = 0.024; ES: 0.40), and COD_LOW_ (*p* = 0.038; ES: 0.31) following 3 days of interval between the games when compared with 2 days. However, when analyzed relative to effective time, non-significant differences were found. In summary, CPs seem to not affect the match external load, but players performed better in terms of DEC and COD following 3 days of interval when compared to 2 days when analyzed with absolute values.

## 1. Introduction

Futsal is a high-intensity, intermittent team sport characterized by frequent quick and fast movements which impose significant accelerations (ACC), decelerations (DEC), and changes of direction (COD) [1,2,3]. Due to the small court size and the reduced time for play, players perform short (i.e., typically less than 10 m) and high-intensity actions [1,4,5]. In elite futsal teams, players regularly compete in match-congested national and international tournaments, with three matches in four days or one week (i.e., with only one to three days of recovery intervals within the games) [6,7,8,9]. Despite its growing popularity in recent years, research on futsal external match load metrics (e.g., player load [PL] and low- to high-intensity ACC, DEC, and COD), especially during congested periods (CPs), remains limited [10,11,12].

A team’s ability to endure these CPs may play a crucial role in its overall performance [13,14,15]. The limited time between matches may prove insufficient for complete player recovery, potentially resulting in acute and chronic fatigue. Consequently, it can negatively affect match performance (e.g., high-intensity actions) [16,17], increase the risk of injury [13,18], influence match outcomes [19], and adversely affect various physical abilities, including sprinting, agility, and jumping [20,21]. Given this information, understanding the physical match demands during CPs is critical to optimize tailor-made training programs, improve physical performance, and prevent injuries.

A recent study [22] in professional football players found that congested periods seemed to not affect official match locomotor performance. Similarly, a study involving elite futsal players by Ribeiro et al. [12] found that external match load metrics (i.e., total distance covered, high-speed running, ACC, and DEC) increased from the first to the third game of a CP, despite players reporting lower perceived exertion. The authors suggested that playing time could be a key factor mediating the responses during CPs, as confirmed by a study [21] conducted with young futsal players during a 4-day tournament. In that research [21], players with more minutes of playing time exhibited reduced lower-body power (i.e., countermovement jump power) and perceived recovery compared with those with less minutes. Similarly, a recent study by González-Fernández et al. [11] revealed that, during an 11-day training camp comprising five friendly matches, only the sprint, DEC, and ACC performance of young futsal players significantly differed among matches, with the body composition and the physical fitness measures being correlated with match-running demands. In another research, Charlot et al. [23] observed increased internal load during a 4-day tournament but found no significant differences in high-intensity activities (measured by time spent at >80% of resting heart rate) or subjective well-being indices across four matches. However, it is important to highlight that these previous studies [10,12] (1) assessed players’ external load during futsal match-congested tournaments, not during the regular competitive period (e.g., comprising national league and cup fixtures); and (2) did not take into account the interval days between the games (e.g., 2- versus 3-day interval). Consequently, more research is needed to explore external match load during CPs, as well as how different intervals between games impact match demands and recovery in professional futsal players during the in-season.

Therefore, the present study aims to analyze the external match demands during a CP (e.g., three matches in eight days) and examine the differences in match demands based on recovery intervals of two versus three days in professional futsal players. It is hypothesized that players will maintain or exhibit slightly increased external load metrics during the CP, consistent with findings from previous research on professional futsal players [12], where external load (i.e., total distance covered, high-speed running, ACC, and DEC) was progressively increased from the first to the third match in almost all variables, and match demands will remain similar regardless of whether the recovery interval is 2 or 3 days.

## 2. Materials and Methods

### 2.1. Study Design

A retrospective observational study was conducted to assess the external load of professional futsal players. Data were collected using wearable technology (i.e., accelerometers [Catapult Innovation; Melbourne, Australia]) during all official matches of the National Futsal League (LNFS; 1st Division of Spain), Copa del Rey, and the UEFA Futsal Champions League from the 2019–2020 and 2020–2021 seasons. For the purpose of the study, only matches during CPs were analyzed. A CP was considered whenever 3 official matches were played within an 8-day span (i.e., one competitive microcycle). Following this criterion, a total of 15 matches were included in the analysis (Table 1). The matches were categorized based on the number of days between games: 2 days (i.e., 5 occurrences) and 3 days (i.e., 5 occurrences). All weeks before the CP were considered “normal”, as each had only one official competition (i.e., >6 days between matches). According to official rules, the matches lasted 40 min and consisted of two halves of 20 min separated by a 10 min halftime. Throughout the two seasons, 11 out of 15 players from the team were selected by the coaching staff and monitored due to the availability of GPS units. The current study included only in-court players that played >5 min of effective time. The study procedures did not influence or alter the course of the games.

### 2.2. Participants

Eleven elite male futsal players (age: 26.7 ± 3.1 years; body mass: 74.7 ± 5.9 kg; height: 1.78 ± 0.06 m; body fat: 8.8 ± 1.5%) from the same team competing in the LNFS were monitored for this study. All the participants had signed a professional contract with the club and provided consent for data collection and participation in scientific research, on the condition that data would be anonymized. The procedures were approved by the Ethics Committee of the Catholic University of Murcia under code CE072008 and conducted in accordance with the Declaration of Helsinki.

### 2.3. Procedures

External load metrics were recorded using a Catapult Sport Optimeye S5 GPS unit (Catapult Innovation; Melbourne, Australia), equipped with a triaxial accelerometer, gyroscope, and magnetometer. These sensors provided inertial movement data at a sampling rate of 100 Hz, which are valid and reliable [24]. Each device was secured to the upper back of the players using a specialized vest worn under their jerseys. To minimize inter-unit variability, each player consistently used the same device throughout the season. The match-play external load metrics began in the locker room after the warm-up, approximately 10 min before starting the match, and ended before the postgame cool-down. All data were analyzed by Catapult Sport Openfield software version 3.9 (Catapult Innovation; Melbourne, Australia), which transformed the raw data during athlete movement into output variables used to quantify the movement experience. Finally, all the data were exported to a Microsoft Excel ^TM^ spreadsheet for further analysis.

This study included total PL, PL per minute (PL·min^−1^), ACC, DEC, and COD. The last three variables were examined across three intensity levels, high (>3.5 m·s^−2^), medium (2.5–3.50 m·s^−2^), and low (<2.5 m·s^−2^) (i.e., the thresholds were defined from the software and used previously in futsal [3]), as well as the total sum of each parameter. PL consists of the sum of the ACC across all axes of the internal tri-axial accelerometer during movement (100 Hz), calculated using a predetermined formula and expressed as an arbitrary unit (a.u). PL·min^−1^ divides the accumulated PL by time, providing an intensity index also expressed as an a.u [25]. Previous studies [26,27] confirmed the validity and reliability of the aforementioned variables.

All data were measured and analyzed using two approaches. The first was the absolute method, which included all periods across the entire match duration and measured players’ external load without accounting for effective playing time. The second was the relative method, which normalized load by dividing the external load by the effective playing time.

### 2.4. Statistical Analysis

A statistical package (Jamovi, version 1.8, 2021) was used for the statistical analysis. A descriptive statistic with mean ± standard deviation was performed for all the variables. Linear mixed models were constructed to examine differences in external load variables amongst games, accounting for individual repeated measures. In the linear mixed model, game level (three levels) was used as a fixed effect and player as a random effect with a random intercept and fixed slope. Moreover, the day interval level (two levels) was used as a fixed effect and player as a random effect with a random intercept and fixed slope. Pairwise comparisons were performed using the Bonferroni post hoc analysis if a significant interaction effect was found amongst games. Cohen’s d ES and 95% confidence intervals (CIs) were determined and interpreted as follows: <0.2, trivial; 0.20–0.59, small; 0.60–1.19, moderate; 1.2–1.99, large; and ≥2.0, very large [28]. A significance level was set at *p* < 0.05.

## 3. Results

Table 2 and Table 3 describe the data for selected external load variables amongst games. Concerning the external match load during every CP (Table 2 and Table 3), non-significant differences were found for all the variables (PL, PL·min^−1^, total ACC, DEC, and COD across the three intensities) (*p* = 0.108–0.995; ES: 0.01–0.40), which were even normalized by the effective time (*p* = 0.338–0.886; ES: 0.09–0.28).

Table 4 and Table 5 describe the data for selected external load variables considering the interval days between the games. Results revealed that players performed significantly more DEC_HI_ (*p* = 0.030; ES: 0.48), COD_TOTAL_ (*p* = 0.028; ES: 0.33), COD_MED_ (*p* = 0.024; ES: 0.40), and COD_LOW_ (*p* = 0.038; ES: 0.31) following a 3-day interval when compared to only 2 days (Table 4). However, normalizing the match external load by effective time, non-significant differences (*p* = 0.163–0.997; ES: 0.01–0.31) were found between 2- and 3-day intervals between games.

## 4. Discussion

Understanding the physical match performance of futsal players during congested periods and the effects of varying recovery intervals between matches is critical for developing an effective weekly training plan to optimize training programs and mitigate overuse injury risk. The aims of this study were to quantify the external match load during CPs (i.e., three games in 8 days) and compare the match demands when a different number of interval days existed between matches (i.e., 2 vs. 3 days) in professional futsal players. The overall results relating to the abovementioned objectives were that CPs seem to not affect the general external match load in professional futsal players. However, the number of recovery days between consecutive matches might be an influential factor. According to the current results, considering absolute values, players performed significantly more DEC_HI_, COD_TOTAL_, COD_MED_, and COD_LOW_ following 3 interval days between the games when compared with 2 days. Nevertheless, no differences were found when normalized by the effective time. With this information, coaches may optimize player management during CPs.

In futsal, mechanical variables such as DEC, ACC, and COD are used to determine the performance of actions with or without a ball [1,3,5]. These variables align with the neuromuscular and biomechanical futsal demands, but also correspond to the movement patterns of players throughout the match. The results from the current study revealed that external load metrics seemed to be maintained throughout the CP, which is in line with previous research [12]. Specifically, Ribeiro et al. [12] found that futsal players who competed for longer periods showed lower external loads and higher internal loads in the first match, with a progressive increase from the first to the third match in almost all variables compared to players who competed for shorter periods. Additionally, Charlot et al. [23] observed an increase in internal load during a 4-day tournament but did not report significant changes in high-intensity activities (defined by time spent above 80% of resting heart rate) or subjective well-being indicators across the matches. This phenomenon could be explained due to futsal’s nature [29,30]. Specifically, the unlimited number of substitutions could affect the players’ capacity to sustain performance over the matches [21,31]. In futsal, playing time has been proposed as a crucial factor influencing responses during CPs, as evidenced by a study [12,21]. From a practical perspective, futsal coaches and practitioners are advised to manage players’ interchange rotations (substitutions) to positively impact players’ recovery and readiness for performance [31].

Furthermore, an important aspect during CPs is the number of days players have to “recover” and prepare for the next game. For instance, in other team sports such as football (i.e., soccer), studies have found that players are fully recovered only after 72 h, whereas at 48 h, some physical capacities are still impaired [32,33]. However, this impairment seems not to reflect the match demands in professional football players [22]. Specifically, a recent study [22] in professional football players found that congested periods seemed to not affect official match locomotor performance. When it comes to futsal, to the authors’ knowledge, this is the first study to investigate the external match load in futsal considering different intervals between games, specifically 2 and 3 days. Herein, the number of interval days was only found to have a significant effect on DEC and COD. Specifically, it is important to highlight that the players performed significantly more DEC_HI_, COD_TOTAL_, COD_MED_, and COD_LOW_ following 3 days of rest between matches when compared to 2 days. From these observations, it appears that despite being ready to play, futsal players increase their readiness and availability to perform at a high level after 3 days of recovery. However, these differences were not observed when the external match load by the effective time was analyzed. Thus, it is suggested that futsal body governance should revise competitive calendars to allow sufficient days of recovery between matches (i.e., at least 3 days), thus ensuring players’ health and performance are optimal [21]. Interestingly, despite the study’s limitations (described below) and the various multidimensional factors that can influence match outcomes, games after 3 days of preparation showed a greater tendency to achieve a positive result (i.e., 100% winning) compared to games with only 2 days of preparation (20% winning) (see Table 1).

Despite the results, it is important to highlight that the present research provides match data based on accelerometry from a total of 15 games (e.g., 5 weeks of a CP) played by professional players, which may restrict the generalizability of the results to other futsal teams and competitions. However, the sample comprised games from different competitions over the seasons. Additional information is also required regarding the travel distance and the conditions of recovery and training before the matches. Moreover, it is limited by reporting only the external load without considering distance covered at different velocities and, more importantly, players’ internal responses (e.g., heart rate, perceived exertion, and/or recovery). Lastly, the fact that no physical tests investigating players’ neuromuscular performance (i.e., sprint and COD ability, unilateral/bilateral jumps, and maximal strength) were performed limits our understanding of the specific effects of the CP on physical performance and whether players perform these actions under excessive fatigue or not. Further research about the total training load in the previous days, such as −3, −2, and −1 Match Day, and the variation in the players’ external load is warranted for the futsal community.

## 5. Conclusions

In summary, the competition activity profile of professional futsal players and external match demands seem to be maintained during a CP (i.e., three games in 8 days). Nevertheless, the number of recovery days between consecutive matches should be taken into account. Players performed significantly more DEC_HI_, COD_TOTAL_, COD_MED_, and COD_LOW_ following a 3-day interval between the games when compared to 2 days. In applied settings, futsal coaches and sports practitioners are advised to carefully manage playing time and substitution strategies to mitigate acute and residual neuromuscular fatigue during CPs and, thus, maintain players’ performance. Additionally, futsal governing bodies are advised to give sufficient days of recovery between matches to ensure players’ health and performance are optimal.

## Figures and Tables

**Table 1:** Description of the games and trainings during the analyzed congested periods.

Congested Weeks								
Days	1	2	3	4	5	6	7	8
Week 1	LNFS (*medium*) W (2-1)	Training	Training	Training	RC (*medium*) W (5-1)	Training	Training	LNFS (*low*) W (0-2)
Week 2	LNFS (*low*) W (3-5)	Training	Training	LNFS (*high*) D (1-1)	Training	Training	Training	UFCL (*low*) W (5-0)
Week 3	LNFS (*medium*) W (4-5)	Training	Training	CdR (*medium*) L (3-1)	Training	Training	Training	LNFS (*low*) W (2-1)
Week 4	LNFS (*low*) W (1-5)	Training	Training	Training	LNFS (*low*) W (5-1)	Training	Training	LNFS (*low*) D (4-4)
Week 5	LNFS (*high*) L (4-1)	Training	Training	Training	LNFS (*medium*) W (0-1)	Training	Training	LNFS (*medium*) L (0-3)

CdR: *Copa del Rey* (cup match); D: draw; L: loss; LNFS: *Liga Nacional de Futbol Sala* (league match); RC: *regional cup*; UFCL: *UEFA Futsal Champions League* (European competition match); W: win. High, medium, and low correspond to the level of the opposing team (i.e., based on the classification of the opponent on the corresponding season).

**Table 2:** Comparison of the data for selected external load variables amongst games during the congested periods.

Variables	Congested Period			*p* Value	Effect Size
	1st Game	2nd Game	3rd Game		
Player Load	215 ± 73	223 ± 59	226 ± 73	0.499	0.22
PL·min^−1^	11.2 ± 1.9	11.2 ± 1.2	11.2 ± 1.4	0.818	0.12
ACC_TOTAL_	29 ± 12	30 ± 11	30 ± 12	0.937	0.06
ACC_HI_	4 ± 2	3 ± 3	4 ± 3	0.316	0.28
ACC_MED_	7 ± 4	7 ± 3	7 ± 4	0.911	0.08
ACC_LOW_	19 ± 9	19 ± 8	19 ± 8	0.995	0.01
DEC_TOTAL_	41 ± 18	42 ± 14	45 ± 16	0.671	0.17
DEC_HI_	4 ± 3	4 ± 3	4 ± 2	0.577	0.19
DEC_MED_	11 ± 6	10 ± 5	11 ± 5	0.910	0.08
DEC_LOW_	27 ± 11	28 ± 10	30 ± 12	0.459	0.07
COD_TOTAL_	178 ± 67	191 ± 60	190 ± 82	0.315	0.29
COD_HI_	9 ± 6	11 ± 5	10 ± 6	0.108	0.40
COD_MED_	30 ± 12	33 ± 12	32 ± 15	0.308	0.29
COD_LOW_	139 ± 54	148 ± 47	146 ± 65	0.411	0.25

The data represent the average values across all five weeks. ACC_TOTAL_: total number of accelerations; ACC_HI_: high-intensity acceleration; ACC_MED_: medium-intensity acceleration; ACC_LOW_: low-intensity acceleration; DEC_TOTAL_: total number of decelerations; DEC_HI_: high-intensity deceleration; DEC_MED_: medium-intensity deceleration; DEC_LOW_: low-intensity deceleration; COD_TOTAL_: total number of changes of direction; COD_HI_: high-intensity changes of direction; COD_MED_: medium-intensity changes of direction; COD_LOW_: low-intensity changes of direction.

**Table 3:** Comparison of the data for selected external load variables per minute amongst games during the congested periods.

Variables	Congested Period			*p* Value	Effect Size
	1st Game	2nd Game	3rd Game		
Player Load·min^−1^	11.2 ± 2.05	11.3 ± 1.46	11.2 ± 1.43	0.830	0.11
ACC_TOTAL_·min^−1^	1.52 ± 0.46	1.48 ± 0.41	1.49 ± 0.49	0.886	0.09
ACC_HI_·min^−1^	0.19 ± 0.01	0.21 ± 0.12	0.21 ± 0.11	0.558	0.20
ACC_MED_·min^−1^	0.36 ± 0.21	0.35 ± 0.16	0.33 ± 0.17	0.656	0.17
ACC_LOW_·min^−1^	0.97 ± 0.34	0.93 ± 0.31	0.96 ± 0.34	0.813	0.12
DEC_TOTAL_·min^−1^	2.14 ± 0.68	2.15 ± 0.58	2.26 ± 0.59	0.685	0.16
DEC_HI_·min^−1^	0.19 ± 0.12	0.21 ± 0.14	0.21 ± 0.09	0.466	0.23
DEC_MED_·min^−1^	0.55 ± 0.28	0.52 ± 0.21	0.54 ± 0.21	0.511	0.22
DEC_LOW_·min^−1^	1.41 ± 0.43	1.41 ± 0.46	1.51 ± 0.49	0.475	0.23
COD_TOTAL_·min^−1^	9.33 ± 2.35	9.71 ± 1.97	9.32 ± 2.38	0.362	0.27
COD_HI_·min^−1^	0.48 ± 0.23	0.54 ± 0.24	0.51 ± 0.24	0.387	0.26
COD_MED_·min^−1^	1.60 ± 0.56	1.66 ± 0.45	1.64 ± 0.57	0.883	0.09
COD_LOW_·min^−1^	7.25 ± 1.76	7.50 ± 1.57	7.17 ± 1.85	0.338	0.28

The data represent the average values across all five weeks. ACC_TOTAL_: total number of accelerations; ACC_HI_: high-intensity acceleration; ACC_MED_: medium-intensity acceleration; ACC_LOW_: low-intensity acceleration; DEC_TOTAL_: total number of decelerations; DEC_HI_: high-intensity deceleration; DEC_MED_: medium-intensity deceleration; DEC_LOW_: low-intensity deceleration; COD_TOTAL_: total number of changes of direction; COD_HI_: high-intensity changes of direction; COD_MED_: medium-intensity changes of direction; COD_LOW_: low-intensity changes of direction.

**Table 4:** Comparison of the data for selected external load variables considering the number of interval days between consecutive games.

Variables	Interval Days Between Games		*p* Value	Effect Size
	2	3		
Player Load	213 ± 65	237 ± 66	0.056	0.35
PL·min^−1^	11.2 ± 1.2	11.2 ± 1.4	0.899	0.00
ACC_TOTAL_	29 ± 11	31 ± 12	0.356	0.17
ACC_HI_	4 ± 2	5 ± 3	0.144	0.26
ACC_MED_	7 ± 3	7 ± 4	0.731	0.06
ACC_LOW_	19 ± 8	19 ± 8	0.605	0.11
DEC_TOTAL_	42 ± 16	45 ± 13	0.305	0.22
**DEC_HI_**	**4 ± 2**	5 ± 3 *	**0.030**	**0.48**
DEC_MED_	10 ± 5	11 ± 5	0.275	0.23
DEC_LOW_	28 ± 12	29 ± 9	0.709	0.09
**COD_TOTAL_**	**179 ± 72**	203 ± 70 *	**0.028**	**0.33**
COD_HI_	10 ± 5	11 ± 5	0.115	0.20
**COD_MED_**	**31 ± 12**	36 ± 15 *	**0.024**	**0.40**
**COD_LOW_**	**138 ± 57**	156 ± 54 *	**0.038**	**0.31**

The data represent the average values across all five weeks. * *p* < 0.05. ACC_TOTAL_: total number of accelerations; ACC_HI_: high-intensity acceleration; ACC_MED_: medium-intensity acceleration; ACC_LOW_: low-intensity acceleration; DEC_TOTAL_: total number of decelerations; DEC_HI_: high-intensity deceleration; DEC_MED_: medium-intensity deceleration; DEC_LOW_: low-intensity deceleration; COD_TOTAL_: total number of changes of direction; COD_HI_: high-intensity changes of direction; COD_MED_: medium-intensity changes of direction; COD_LOW_: low-intensity changes of direction. Variables in bold correspond to the ones in which significant differences were found.

**Table 5:** Comparison of the data for selected external load variables per minute considering the number of interval days between consecutive games.

Variables	Interval Days Between Games		*p* Value	Effect Size
	2	3		
Player Load·min^−1^	11.2 ± 1.28	11.3 ± 1.60	0.951	0.03
ACC_TOTAL_·min^−1^	1.52 ± 0.46	1.45 ± 0.44	0.483	0.15
ACC_HI_·min^−1^	0.20 ± 0.09	0.22 ± 0.13	0.466	0.13
ACC_MED_·min^−1^	0.35 ± 0.16	0.32 ± 0.18	0.316	0.20
ACC_LOW_·min^−1^	0.98 ± 0.35	0.91 ± 0.30	0.368	0.19
DEC_TOTAL_·min^−1^	2.21 ± 0.58	2.20 ± 0.59	0.758	0.01
DEC_HI_·min^−1^	0.20 ± 0.09	0.23 ± 0.13	0.151	0.31
DEC_MED_·min^−1^	0.53 ± 0.21	0.53 ± 0.20	0.997	0.01
DEC_LOW_·min^−1^	1.48 ± 0.47	1.43 ± 0.48	0.459	0.10
COD_TOTAL_·min^−1^	9.35 ± 2.10	9.69 ± 2.29	0.275	0.15
COD_HI_·min^−1^	0.52 ± 0.22	0.54 ± 0.27	0.635	0.06
COD_MED_·min^−1^	1.59 ± 0.46	1.72 ± 0.57	0.163	0.25
COD_LOW_·min^−1^	7.23 ± 1.75	7.43 ± 1.69	0.379	0.11

The data represent the average values across all five weeks. ACC_TOTAL_: total number of accelerations; ACC_HI_: high-intensity acceleration; ACC_MED_: medium-intensity acceleration; ACC_LOW_: low-intensity acceleration; DEC_TOTAL_: total number of decelerations; DEC_HI_: high-intensity deceleration; DEC_MED_: medium-intensity deceleration; DEC_LOW_: low-intensity deceleration; COD_TOTAL_: total number of changes of direction; COD_HI_: high-intensity changes of direction; COD_MED_: medium-intensity changes of direction; COD_LOW_: low-intensity changes of direction.

## Data Availability

The datasets generated and/or analyzed during the present study are available from the corresponding author on request. The data are not publicly available due to privacy restrictions.
